# Antibody-based cancer immunotherapy by targeting regulatory T cells

**DOI:** 10.3389/fonc.2023.1157345

**Published:** 2023-04-27

**Authors:** Quanxiao Li, Jun Lu, Jinyao Li, Baohong Zhang, Yanling Wu, Tianlei Ying

**Affiliations:** ^1^ Key Laboratory of Medical Molecular Virology (MOE/NHC/CAMS), Shanghai Frontiers Science Center of Pathogenic Microorganisms and Infection, Shanghai Institute of Infectious Disease and Biosecurity, Shanghai Engineering Research Center for Synthetic Immunology, School of Basic Medical Sciences, Shanghai Medical College, Fudan University, Shanghai, China; ^2^ Auckland Bioengineering Institute, University of Auckland, Auckland, New Zealand; ^3^ Xinjiang Key Laboratory of Biological Resources and Genetic Engineering, College of Life Science and Technology, Xinjiang University, Urumqi, Xinjiang, China; ^4^ Engineering Research Center of Cell and Therapeutic Antibody, Ministry of Education, School of Pharmacy, Shanghai Jiao Tong University, Shanghai, China

**Keywords:** antibody, cancer, immunotherapy, regulatory (Treg) cell, immune checkpoint inhibitors

## Abstract

Regulatory T cells (Tregs) are among the most abundant suppressive cells, which infiltrate and accumulate in the tumor microenvironment, leading to tumor escape by inducing anergy and immunosuppression. Their presence has been correlated with tumor progression, invasiveness and metastasis. Targeting tumor-associated Tregs is an effective addition to current immunotherapy approaches, but it may also trigger autoimmune diseases. The major limitation of current therapies targeting Tregs in the tumor microenvironment is the lack of selective targets. Tumor-infiltrating Tregs express high levels of cell surface molecules associated with T-cell activation, such as CTLA4, PD-1, LAG3, TIGIT, ICOS, and TNF receptor superfamily members including 4-1BB, OX40, and GITR. Targeting these molecules often attribute to concurrent depletion of antitumor effector T-cell populations. Therefore, novel approaches need to improve the specificity of targeting Tregs in the tumor microenvironment without affecting peripheral Tregs and effector T cells. In this review, we discuss the immunosuppressive mechanisms of tumor-infiltrating Tregs and the status of antibody-based immunotherapies targeting Tregs.

## Introduction

Regulatory T cells (Tregs) constitute 1–2% of the peripheral CD4^+^ lymphocyte population, which express high levels of the regulatory transcription factor forkhead box protein 3 (FOXP3), CD25 (IL-2Rα), cytotoxic T lymphocyte (CTL)-associated antigen-4 (CTLA4), glucocorticoid induced tumor necrosis factor receptor (GITR), and CD39 (1, 2). Tregs intrinsically function to maintain immune homeostasis, support self-tolerance, and downregulate excessive immune responses against auto-antigens *via* different mechanisms (3). They are produced by the thymus and then exported to the periphery. In healthy individuals, Tregs suppress the potential self-reactive T cells through active regulation.

Despite critical importance, the immunosuppressive activities of Tregs are not desired in the tumor microenvironment (TME). Tregs are considered to play key roles in tumor immune escape by hampering tumor-specific T-cell responses and promoting tumor growth (4). Changes of nutrient composition, oxygen availability, and cytokines and chemokines released in TME favor Treg infiltration and effector T cell (Teff) exhaustion (5). This correlation also exists in patients that do not respond to immune checkpoint inhibitor (ICI) treatment. Hence, Treg infiltration in TME has been appreciated as a biomarker and a predictive factor for tumor progression and therapy response (6, 7). Removal of Tregs can evoke effective antitumor immunity in tumor-bearing animals (8). In humans, cancer immunotherapy by depleting Tregs is under clinical trial. Here, we review Treg biology, immunosuppressive mechanisms, and interactions in the TME context. We also summarize the current antibody-based immunotherapies and their efficacy of targeting Tregs in the treatment of tumors.

## Phenotype of Tregs

Tregs are categorized in three distinct subsets according to their sites of development. The thymic Tregs (tTregs) are developed in the thymus from the precursors of CD4^+^ helper T (Th) cells, which show an enrichment of T-cell receptors (TCRs) with high affinity for self-antigens (9, 10). Peripheral Tregs (pTregs) differentiate from mature CD4^+^ Th cells in the periphery upon encounter with antigens. A third Treg type is known as induced Tregs (iTregs), which are developed from naïve T cells and require antigen stimulation in the presence of transforming growth factor-beta (TGF-β) and interleukin-2 (IL-2) (11).

By their functions, Tregs can be further specified in two main subsets, central Tregs (cTregs) and effector Tregs (eTregs), in peripheral lymphoid organs (12). cTregs are thymic emigrants that have not been activated, which exhibit a naïve phenotype (CD45RA^+^ FOXP3^lo^) with low suppressive activity. They are enriched in lymphoid tissues where they express lymphoid-tissue homing molecules involved in trafficking to secondary lymphoid organs (such as CD62L and CCR7) and are dependent on IL-2 to maintain their state of rest (13, 14). After antigen engagement by TCRs, cTregs differentiate into eTregs (CD45RA^-^ FOXP3^hi^), which upregulate activation marker CD44, effector molecules CTLA4, GZMB and KLRG1, and immunosuppressive cytokines (15, 16). By upregulating these molecules, eTregs further direct the migration and localization to non-lymphoid peripheral sites in response to specific stimuli (17).

**Figure 1:**
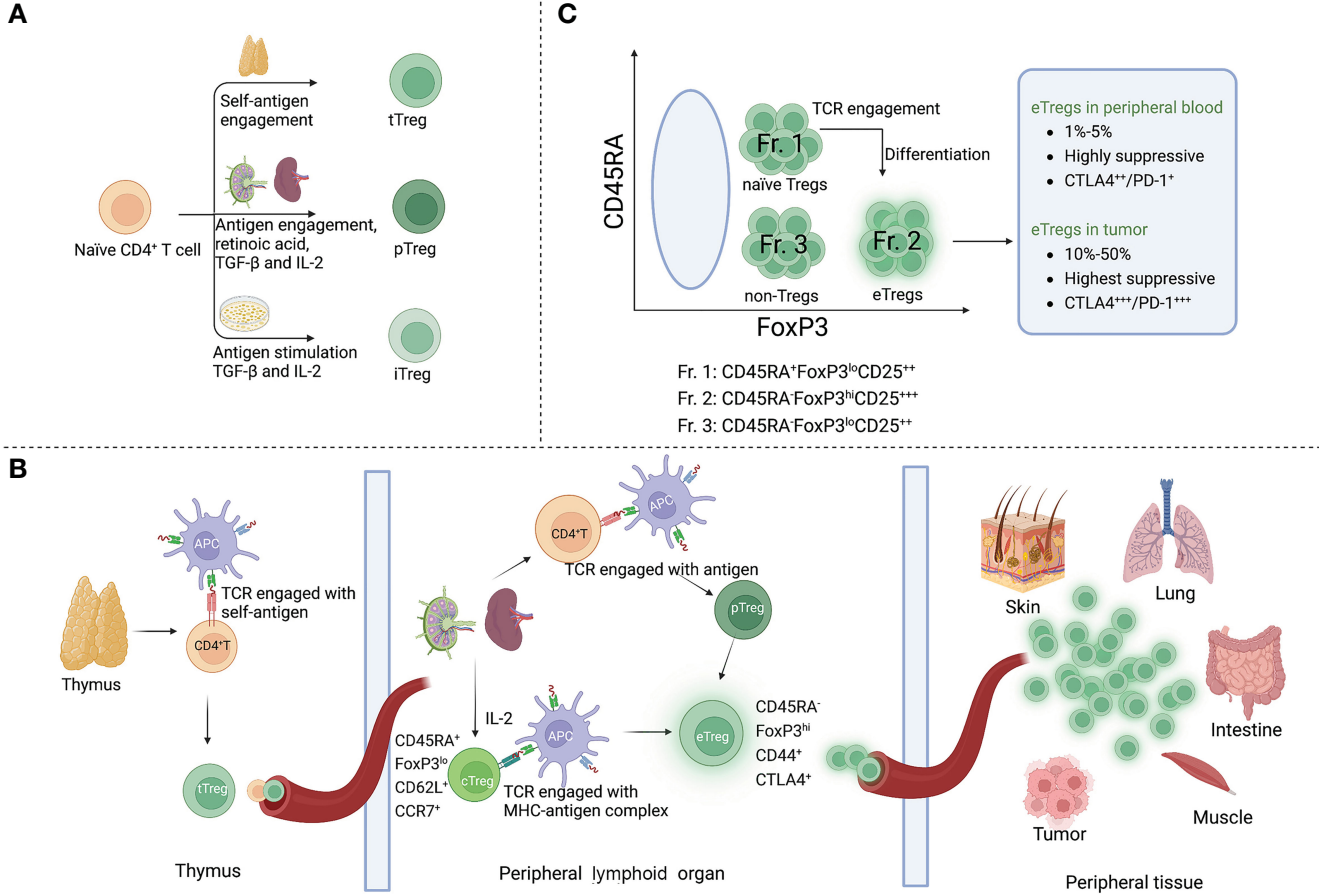
Phenotype and development of Tregs. **(A)** There are three Treg subtypes according to the sites of development: tTregs are developed in the thymus from the precursors of CD4^+^ T cells; pTregs are differentiated from mature CD4^+^ Th cells in the periphery upon encounter with antigens and other factors (IL-2, TGF-β, and retinoic acid); iTregs are developed from naïve T cells and require antigen stimulation in the presence of TGF-β and IL-2. **(B)** Development of cTregs and eTregs: tTregs migrate to peripheral lymphoid organs after thymus development and become cTregs, maintaining a naïve phenotype in the presence of IL-2 (CD45RA^+^, FOXP3^lo^, CD62L^+^, CCR7^+^). When cTregs and pTregs encounter TCR stimulation, they further differentiate into eTregs with expression of activation molecules CD44 and CTLA4. eTregs migrate and localize to non-lymphoid peripheral sites in response to specific stimuli. **(C)** Subfractions of human FOXP3^+^ Tregs: Fr. 1, CD45RA^+^ FOXP3^lo^ CD25^++^ naïve Tregs; when naïve Tregs engage with antigens, they differentiate into Fr. 2 eTregs (CD45RA^-^ FOXP3^hi^ CD25^+++^); Fr. 3, non-Tregs, defined by CD45RA^-^ FOXP3^lo^ CD25^++^ cells.

Some conventional T cells (Tconvs) also express FOXP3 at low levels in the blood without exhibiting suppressive activity (18). To distinguish between suppressive and non-suppressive subtypes, CD4^+^FOXP3^+^ Tregs could be classified in three categories in the blood of healthy individuals by their different expression levels of FOXP3 (and CD25) and CD45RA. Naïve/resting Tregs, defined by CD45RA^+^ FOXP3^lo^CD25^++^ cells (Fraction (Fr.) 1, see **Figure 1**), derive from thymus with weak suppressive activity. After TCR stimulation in the draining lymph node, naïve Tregs proliferate and differentiate into highly suppressive and terminally differentiated eTregs (Fr. 2), defined by CD45RA^−^FOXP3^hi^CD25^+++^. In general, the frequency of eTregs of CD4^+^ T cells in humans is 1%-5% in the peripheral blood but approximately 10%-50% in most TME (19). Non-Tregs (Fr. 3) are defined by FOXP3^lo^CD45RA^−^CD25^++^ cells (18).

## Mechanisms of Treg-mediated immunosuppression

**Figure 2:**
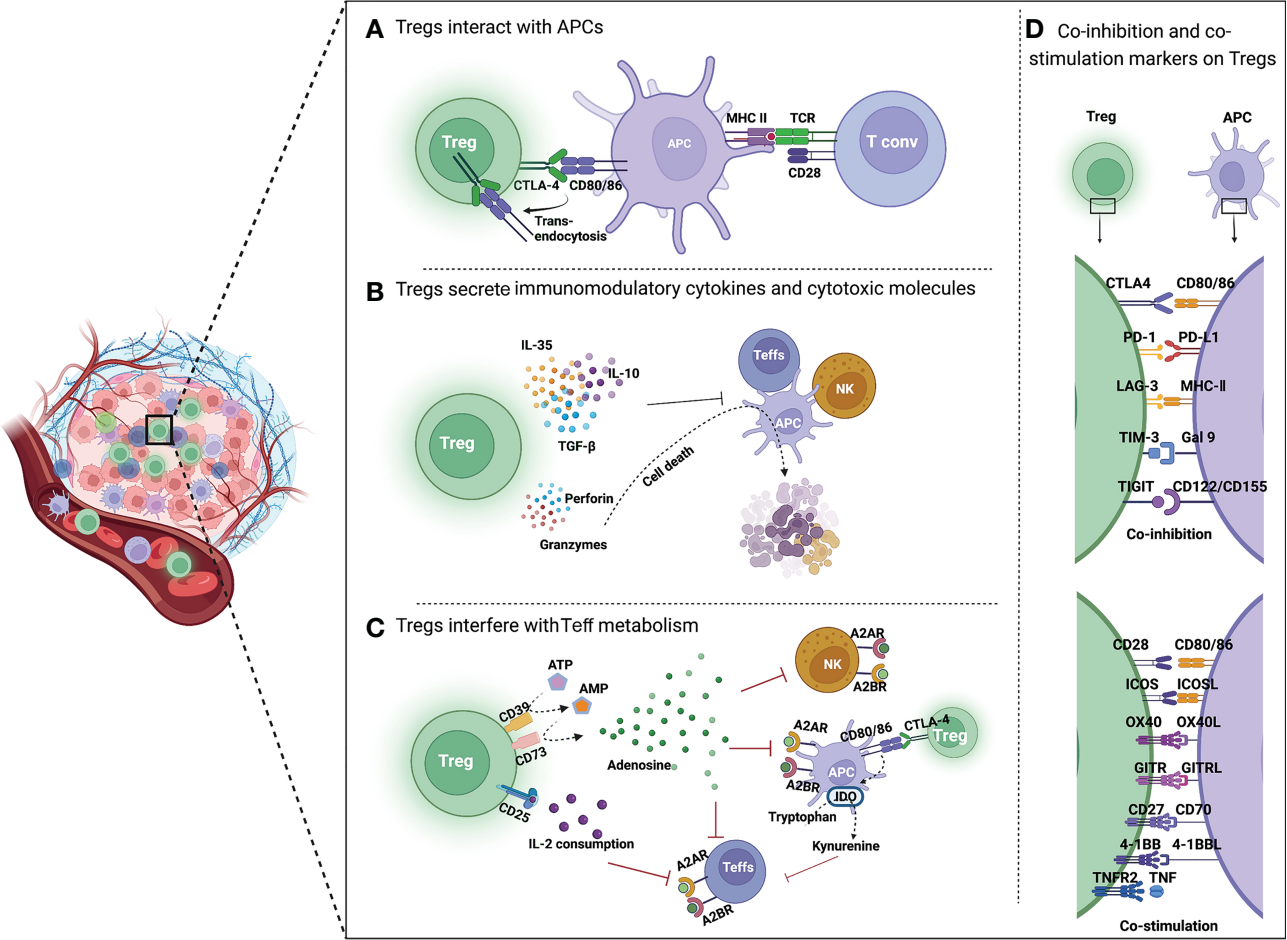
Mechanisms of Treg-mediated immunosuppression in TME. **(A)** Tregs inhibit the function of APCs and Teffs through immune checkpoints. CTLA4 is highly expressed on Tregs, which binds to CD80/86 on APCs, reduces CD80/86 expressed by APCs through trans-endocytosis, and inhibits the activation of T cells by APCs. **(B)** Tregs secrete immunomodulatory cytokines and cytotoxic molecules. Tregs directly inhibit the activation of Teffs, NK cells and APCs by secreting immunosuppressive cytokines such as IL-35, IL-10 and TGF-β. Moreover, Tregs can kill immune cells by directly secreting perforin and granzyme, leading to cell apoptosis. **(C)** Tregs interfere with Teff metabolism. CD39 converts extracellular ATP to generate AMP, which is then cleaved by CD73 to produce immunosuppressive molecular adenosine. Adenosine binds to receptors (A2A or A2B) on the surface of Teffs, APCs and NK cells, resulting in their immunosuppression. CTLA4 induces IDO production by APCs, which can oxidize tryptophan to kynurenine. Tryptophan is an essential amino acid for maintaining T cell activation. **(D)** Co-inhibition and co-stimulation molecules expressed by Tregs and their ligands. Tregs express a range of co-inhibition molecules of immune checkpoints (CTLA4, PD-1, LAG3, TIM3, and TIGIT) and co-stimulation molecules of Ig superfamily (CD28 and ICOS) as well as TNFR superfamily (OX40, GITR, CD27, 4-1BB, and TNFR2) on the surface.

Previous studies have demonstrated that Tregs have different immunosuppressive mechanisms, which can be summarized in three aspects. First, Tregs express immune checkpoint receptors, such as CTLA4, by which they interact with and suppress Teffs and antigen-presenting cells (APCs). Additionally, Tregs secrete immunomodulatory cytokines (IL-10, IL-35, and TGF-β) to repress the immune response and cytotoxic molecules (perforin, granzymes A and B), and directly induce apoptosis of effector immune cells. Moreover, Tregs interfere with the metabolism of effector cells thereby affecting their functions (see **Figure 2**) (20).

### Tregs inhibit the function of APCs and Teffs through immune checkpoints

CTLA4 expressed by Tregs shows higher affinity than CD28 when binding to CD80/86 on APCs, thus preventing Tconv activation through the interaction between CD80/CD86 and CD28. Removal of CD80/CD86 by trans-endocytosis results in impaired co-stimulation of T cells *via* CD28, leading to immune suppressive (21).

PD-1 is a transmembrane immunoinhibitory protein of the CD28 Ig superfamily and plays a major role in tumor immune escape. It is expressed at a low level by Tregs in the blood and upregulated in tumor-infiltrating Tregs (22). Different from CTLA4, Teffs in tumor express PD-1 at baseline or higher levels compared to tumor-infiltrating Tregs, potentially indicating exhaustion status. PD-1 engages with its ligands PD-L1/PD-L2 expressed on activated APCs to inhibit T-cell proliferation, survival, and effector functions (23) and induce apoptosis of tumor-specific T cells (24). The regulation and compartmentalization of PD-1 discriminate CD4^+^CD25^+^ resting Tregs from activated T cells. PD-1 signaling pathway is also important for the maintenance of the suppressive capacity of Tregs (25). PD-1 blockade enhanced PD-1^+^ Treg cell-mediated immunosuppression (26), and PD-1 expression in Tregs could be regulated by lactic acid in the highly glycolytic TME (27).

### Tregs secrete immunomodulatory cytokines and cytotoxic molecules

Tregs directly suppress the activity of T and B lymphocytes, dendritic cells (DCs), and macrophages by secreting anti-inflammatory soluble cytokines such as TGF-β, IL-10, and IL-35 (28, 29).

IL-10 secretion by Tregs impedes antigen-presenting effect of DCs. This effect was restored by anti-IL-10 treatment, and antigen-specific Tregs from IL-10^−/−^ mice failed in decreasing antigen presentation of DCs (11). IL-10 can also downregulate MHC-II expression on the surface of mononuclear cells and reduce their antigen-presenting function, which impairs T-lymphocyte activity (11). Adaptive plasticity of IL-10^+^ and IL-35^+^ Treg cells cooperatively promotes tumor T cell exhaustion (28).

TGF-β can regulate the expression of FOXP3. iTregs rapidly lost the ability to express FOXP3 in the absence of TGF-β *in vitro*. TGF-β is important for Treg-induced inhibition of granule exocytosis and cytolytic function of CTLs (30). TGF-β1 is mainly secreted by Tregs. Tregs can express the docking receptor glycoprotein A repetitions predominant (GARP) and αv integrins that activate the cytokines, which triggers differentiation of other cell subsets, including T helper 17 (Th17) cells (29). IL-10 and TGF-β can also induce regulatory gamma-delta T cells, which is an innate-like T-cell population with a suppressive phenotype (31).

IL-35, a member of the IL-12 family, is preferentially secreted by mouse and human Tregs (28). IL-35 can induce Tconv conversion into suppressive CD4^+^ FOXP3^−^ Treg population (32). In an IL-35-reporter mouse model, researchers found that the tumor was infiltrated with many IL-35^+^ Tregs, and tumor growth in both humans and mice was suppressed by neutralizing IL-35 with an antibody. Additionally, IL-35 limits antigen-specific antitumor T-cell responses by downregulating interferon-gamma (IFNγ) and tumor necrosis factor alpha (TNFα) secreted by CTLs. IL-35 also promotes the expression of exhaustion markers PD-1, TIM3, and LAG3 on T cells in TME (33).

In addition to secreting immunosuppressive cytokines, Tregs may directly kill APCs that express the target antigen by secreting perforin (34), while granzymes A and B induce apoptosis in target cells such as CD4^+^ and CD8^+^ T cells, monocytes, and DCs (35). Alternatively, Tregs can induce T cell apoptosis through TRAIL-DR5 pathway or *via* expression of galectin-1 (36) or FasL (37).

### Tregs interfere with Teff metabolism

Tregs can suppress immune-cell activation through metabolic intermediates, including extracellular production of adenosine (38) and indoleamine 2,3-dioxygenase (IDO) (39).

CD39 is an ectonucleotidase overexpressed on the Tregs, which contributes to the conversion of adenosine triphosphate (ATP) to adenosine (40). Adenosine binds to the A2A receptor (A2AR) or A2B receptor (A2BR) on APCs, Teffs, and NK cells, resulting in immunosuppression. Apoptotic Tregs in TME will release large amounts of adenosine, which results in stronger suppression of antitumor immunity (41).

In addition, CTLA4 induces APCs to produce IDO, an essential enzyme in the kynurenine pathway of tryptophan metabolism, which converts tryptophan to kynurenine and leads to “death by starvation” of Teffs with cell cycle arrest (42, 43). IDO expression may also lead to the formation of a tolerant phenotype of IDO-expressing cells themselves (44). This is supported by *in vitro* studies, which showed that tryptophan consumption leads to inhibition of the immunoregulatory kinases mammalian target of rapamycin (mTOR) and protein kinase C, as well as induction of autophagy and Treg activation (45).

### Other mechanisms

CD25 is the high affinity, alpha-chain of the heterotrimer IL-2 receptor (46). High expression of CD25 in Tregs may result in depletion of IL-2 from the surrounding microenvironment, which is detrimental to Teffs that rely on IL-2 for proliferation and activation (19, 47).

Tregs are also able to inhibit the cytotoxicity of NK cells through a cell-cell contact-dependent mechanism (48). In addition, Tregs directly reduce the activation of monocytes and macrophages through Fas/FasL interaction, resulting in decreased cytokine secretion and expression of stimulatory molecules, and induced monocyte apoptosis (49).

## Antibody-based immunotherapies targeting tumor-infiltrating Tregs

At present, antibodies are used as a rational approach to targeting tumor-infiltrating Tregs, and the involved mechanisms are to affect the immunosuppressive effect of Tregs in tumors by blocking or depleting Tregs. **Table 1** summarizes some of the antibody-based immunotherapies that have been approved by Food and Drug Administration (FDA) or are currently under clinical investigation.

**Table 1:** Treg-targeting antibody drugs approved by FDA or in clinical investigation.

Target	Drug name	IgG Type blocking/depletion/agonist	Cancer type	Company	Year of approved/clinical phase
CTLA4	Ipilimumab	IgG1 blocking/depletion	Liver, non-small-cell lung cancer, mesothelioma, renal cell carcinoma	Bristol-Myers Squibb	2011
	Tremelimumab, combination with durvalumab (anti-PD-L1)	IgG2 blocking	Hepatocellular carcinoma	AstraZeneca	2022
TIGIT	Tiragolumab	IgG1 blocking	Breast, cervical, esophageal, gastric, liver, lung, rectal cancer	Roche	Phase 2/3 NCT04300647
	Ociperlimab	IgG1 blocking	Cervical, lung, squamous cell cancer	Beigene	Phase 2 NCT04693234
	Vibostolimab	IgG1 blocking	Triple negative breast, colorectal, endometrial, esophageal, gastrointestinal, stomach, haematological, head and neck, lung, ovarian, prostate, hepatocellular carcinoma, malignant melanoma	Merck Sharp Dohme	Phase 2 NCT04305041
	Domvanalimab	IgG1 blocking	Glioblastoma, non-small-cell lung cancer	Arcus Biosciences	Phase 2 NCT05130177
GITR	BMS986156	IgG1 agonist	Liver, lung cancer	Bristol-Myers Squibb	Phase 1/2 NCT02598960
	Ragifilimab	IgG1 agonist	Glioblastoma, head and neck cancer	Agenus	Phase 2 NCT04225039
	REGN-6569	– agonist	Advanced solid tumor, head and neck tumor, squamous cell carcinoma	Regeneron Pharmaceuticals	Phase 1 NCT04465487
	MK-4166	IgG1 agonist	Advanced solid tumor	Merck Sharp Dohme	Phase 1 NCT02132754
	GWN-323	IgG1 agonist	Advanced solid tumor, lymphoma	Novartis	Phase 1 NCT02740270
OX40	BMS986178	IgG1 agonist	B-cell lymphoma	Bristol-Myers Squibb	Phase 1/2 NCT02737475
	GSK3174998	IgG1 agonist	Head and neck cancer, multiple myeloma	GlaxoSmithKline	Phase 1/2 NCT04126200
	INCAGN1949	IgG1 agonist	Unspecified solid cancer	Agenus	Phase 1/2 NCT02923349
	Ivuxolimab	IgG2 agonist	Breast cancer, acute myelogenous leukemia, squamous cell, renal cell carcinoma	Pfizer	Phase 2 NCT03971409
CD27	Varlilumab	IgG1 agonist	Glioblastoma, haematological lymphoma, prostate cancer, malignant melanoma, renal cell carcinoma	Celldex Therapeutics	Phase 2 NCT04941287
	GEN-1053	– agonist	Solid tumor	Genmab	Phase 1/2 NCT05435339
	Boserolimab	– agonist	Advanced solid tumor, lung cancer	Merck Sharp Dohme	Phase 2 NCT04165096
TNFR2	BI-1808	IgG1 blocking	Lymphoma, cutaneous T-cell cancer	BioInvent	Phase 1/2 NCT04752826
	LBL019	– –	Unspecified solid cancer	Nanjing Leads Biolabs	Phase 1/2 NCT05223231
CD25	Camidanlumab tesirine	IgG1 ADC depletion	Leukemia, advanced solid tumor, burkitts lymphoma, colon tumor, cutaneous T-cell lymphoma, follicle center lymphoma	ADC Therapeutics	Phase 2 NCT04052997
	RG-6292	– depletion	Advanced solid tumor	Roche	Phase 1 NCT04158583
	RM-1995	– ADC depletion	Head and neck tumor, Squamous cell carcinoma	Rakuten Medical	Phase 1 NCT05220748
CCR4	Mogamulizumab	IgG1 depletion	Adult T-cell lymphoma, cutaneous T-cell lymphoma, peripheral T-cell lymphoma, sezary syndrome	Kyowa Kirin	2018
CCR8	BMS-986340	– depletion	Metastatic colorectal, esophageal, head and neck, stomach, non-small-cell lung cancer	Bristol-Myers Squibb	Phase 1/2 NCT04895709
	GS-1811	– depletion	Advanced solid tumor	Gilead Sciences	Phase 1 NCT05007782
	S-531011	IgG1 depletion	Adenocarcinoma, breast, Head and neck cancer, renal cell carcinoma, squamous cell carcinoma	Shionogi & Co	Phase 1/2 NCT05101070
	LM-108	– depletion	Advanced solid tumor	LaNova Medicines	Phase 1/2 NCT05199753
	BAY-3375968	IgG1 depletion	Breast, head and neck, non-small-cell lung cancer, squamous cell carcinoma, melanoma	Bayer	Phase 1 NCT05537740

### Targeting immune checkpoint molecules on Tregs

Since intratumoral Tregs upregulate ICIs, they are a key target for ICIs (50). The main targets of current immune checkpoint therapies include CTLA4, LAG3, TIM3, TIGIT, and IDO.

*CTLA4*

The anti-CTLA4 humanized IgG1 monoclonal antibody ipilimumab was approved by FDA in 2011 for the treatment of advanced melanoma, yet its therapeutic mechanism is still controversial. Previous studies have generally suggested that ipilimumab acts by inhibiting naïve T cell-intrinsic negative regulatory signaling from interacting with CD80/86-CTLA4, or blocking the immune checkpoint thereby activating effector cells. In human tumors, ipilimumab induces intratumoral CD4^+^ and CD8^+^ Teff infiltration without Treg depletion in TME (51). However, preclinical studies in mouse models revealed that the antitumor efficacy of ipilimumab was dependent on the depletion of CTLA4^+^ Tregs in tumors through antibody-dependent cellular cytotoxicity (ADCC) (52–54). CD16-V158F single-nucleotide polymorphisms was associated with higher response rates in inflamed or highly infiltrated tumors in patients with advanced melanoma. These results illustrated that at least in part, anti-CTLA-4 engage with FcγRs and depletion of Tregs (55).

Another anti-CTLA4 antibody tremelimumab, an IgG2 monoclonal antibody without ADCC activity, was approved in 2015 by FDA as an “orphan drug” for treating malignant mesothelioma. In 2022, tremelimumab was approved to treat unresectable hepatocellular carcinoma in combination with durvalumab, an anti-PD-L1 antibody (56).

*TIGIT*

Similar to other co-inhibitory molecules, TIGIT is highly expressed on Tregs (57). There are three ligands of TIGIT, CD155, CD112, and CD113, which are expressed on tumor cells and APCs. TIGIT competitively inhibits binding of CD226 to CD112 and CD155, leading to reduced T-cell activation and proliferation (58).

*In vitro* experiments identified FOXP3^+^ Tregs expressing TIGIT as a distinct Treg subset that specifically suppresses pro-inflammatory Th1 and Th17 cells compared to TIGIT^−^ Tregs (59). Adoptive transfer experiments using TIGIT^−/−^ Tregs exhibited tumor inhibition and enhanced pro-inflammatory cytokine production by CD8^+^ tumor-infiltrating lymphocytes (TILs) (60), which confirmed that TIGIT inhibits anti-tumor immunity predominantly by regulating Treg function.

In 2021, the anti-TIGIT antibody tiragolumab was approved by FDA as “breakthrough therapy designation” to treat patients with non-small-cell lung cancer in combination with anti-PD-L1 antibody atezolizumab. Currently, various TIGIT monoclonal antibodies are being evaluated in clinical trials as therapeutic agents for refractory solid tumors, either as single drugs or in combination with other agents.

### Targeting tumor necrosis factor receptor (TNFR) superfamily

In addition to co-inhibitory immune checkpoint molecules, tumor-infiltrating Tregs also express TNFRs on the surface, such as glucocorticoid-induced TNFR-related protein (GITR), OX40, 4-1BB, inducible co-stimulator (ICOS), TNFR superfamily member 1B (TNFR2), and CD27. Most of them are co-stimulatory receptors that play essential roles in many facets of immune response such as T-cell-antigen priming, expansion, survival, differentiation, and effector functions (59). Agonistic antibodies against these receptors have been demonstrated effective in inhibiting tumor progression in preclinical mouse model.

*GITR*

GITR is a co-stimulatory immune modulating receptor expressed on various immune cell subsets, with particularly high expression on Tregs (61, 62). In mature Tregs, FOXP3 promotes high-level GITR expression (63). GITR activation by its ligand GITRL in CD4^+^ Teffs and CTLs increases cell proliferation and effector function. GITR stimulation in Tregs leads to instability and decreased suppressive function and depletion of Tregs (64). Targeting GITR in Tregs using an agonistic antibody promotes Treg differentiation into Teffs, alleviates Treg-mediated suppression of antitumor immune response, and induces potent antitumor effector cells in glioblastoma (65). A pan-tumor study demonstrated GITR expression variability on tumor-infiltrating Tregs and lymphocytes across tumor types and suggested that patients with non-small-cell lung cancer, renal cell carcinoma, and melanoma may mostly benefit from anti-GITR therapies (66).

DTA-1, a rat IgG2a antibody targeting GITR, was validated for antitumor effect in multiple mouse tumor models including B16, CT26, and MC38. Analysis of TIL subsets after DTA-1 injection found that DTA-1 could reduce the proportion of Tregs in tumors and promote tumor regression. When the effect of ADCC was abolished by N297A mutation of Fc fragment, tumor regression was inhibited (67). This phenomenon indicated that the anti-GITR antibody plays an antitumor role by killing Teffs through Fc-mediated ADCC effect.

Currently, several anti-GITR antibodies are being investigated in early-phase clinical trials. One of them, BMS-986156, has shown to be well tolerated with no dose-limiting toxicities in Phase I/IIa alone or in combination with nivolumab to treat advanced malignant solid neoplasm (68). In addition, a humanized GITR monoclonal antibody MK-4166 was selected to bind to an epitope analogue of DTA-1, which reduced both Treg level and suppressive phenotype while enhancing effector responsiveness in preclinical research (69). However, recent studies on GITR agonists MK-4166, BMS-986156 and GWN323 showed that although they were well tolerated in patients with advanced solid tumors, objective responses were not observed with monotherapy but only in combination treatment with ICIs. The possible reason is that the proportion of Tregs in TME is low (<1% in patients with GWN323 treatment) (70), and only targeting Tregs cannot achieve a therapeutic effect.

*OX40*

OX40 is expressed on the TIL surface of various tumor tissues (71). Murine Tregs constitutively express OX40 and human Tregs upregulate OX40 upon activation (72, 73). OX40 signaling also impacts the generation of Tregs by strongly antagonizing TGF-β-driven FOXP3 mRNA and antigen mediated-conversion of naïve T-cells into FOXP3^+^ Tregs (72). OX40 signaling inhibits FOXP3 expression and Tregs induction through two distinct molecular pathways. OX40 can upregulate BATF3 to produce a closed chromatin configuration that is dependent on Sirt1/7. OX40 can also activate the AKT-mTOR pathway to mediate phosphorylation and nuclear exclusion of the transcription factor Foxo1 (74).

Currently, there are no anti-OX40 antibodies approved by FDA for clinical use, but several are under investigation, such as BMS986178, GSK3174998, INCAGN1949 and Ivuxolimab. BMS-986178 ± nivolumab and/or ipilimumab appeared to have a controllable safety feature, but no clear therapeutic effect was found (75). Moreover, INCAGN01949, a fully human IgG1κ anti-OX40 agonist monoclonal antibody, was designed to promote tumor immunity by Teff activation and Fcγ receptor-mediated Treg depletion. The preliminary efficacy showed that among 87 patients with colorectal, ovarian, and non-small-cell lung cancers, one patient with metastatic gallbladder cancer achieved a partial response, and 23 patients achieved stable disease. However, INCAGN01949 did not increase T-cell activation or decrease Tregs in the peripheral circulation, nor did it show any significant effect on T-cell subsets in the tumor biopsies (76). Therefore, the mechanism of this antibody targeting OX40 needs to be further investigated. Studies in mice have shown that complete Treg depletion was observed after treatment with an anti-OX40 containing a murine IgG2a Fc domain in the colon carcinoma tumor model CT26, whereas only partial depletion was observed with IgG1 Fc domain or Fc was point mutation that eliminates Fc binding activity. In this study, the ratio of CD8^+^ cells in the TME did not decrease. On the contrary, CD8^+^ T expanded significantly, possibly because the OX40 expression of effector T cells in the tumor microenvironment was much lower than Tregs (77).

*CD27*

CD27 is another member of the TNFR superfamily and a key receptor in T-cell costimulatory (78). By binding to CD70 on APC, CD27 can lead to Th1 and CTL activation and proliferation (79–81). However, the amount of CD27^+^ Tregs is higher than CD27^+^ conventional T cells in mice (82). CD27 expression on human Tregs from the peripheral blood and inflamed synovia has been shown with potent suppressive activity (83, 84). The signaling of CD27 stimulation is partially characterized with TNFR-associated factor (TRAF)-mediated and JNK- and NIK-dependent activation of the NFκB pathway (85). Depleting Tregs in a temporal through a mixed bone marrow chimeric mouse model showed that CD27^+^ Tregs prevent the breakdown of peripheral tolerance and limit antitumor immunity. Furthermore, inhibition of Treg-expressed CD27 acts synergistically with PD-1-checkpoint inhibition to improve CTL-mediated immunity against solid tumors (86), suggesting that CD27 is a useful marker in cancer immunotherapy.

Varlilumab is an IgG1-directed CD27 monoclonal antibody used against a variety of solid tumors. A Phase I study showed that the biologic activity of varlilumab is consistent with CD27-engaged chemokine secretion, T-cell activation, and Treg depletion at all dose levels (87). Two other monoclonal antibodies targeting CD27, GEN-1053 and boserolimab, are being evaluated in Phase I/II clinical trials.

*TNFR2*

Previous studies showed that TNF‐α/TNFR2 signaling pathway induced the activation and expansion of mouse and human Tregs (88, 89) and the subset of TNFR2^+^ Tregs were identified with maximal suppressive activity (90). TNFR2 plays a critical role in regulating FOXP3 expression and maintaining the phenotypic and functional stability of Tregs in an inflammatory environment (91). In lung cancer patients, 90% of Tregs in the peripheral blood were TNFR2-positive with highly expressed CTLA4, indicating a strong suppressive phenotype. Compared to TNFR2^−^ Tregs, TNFR2^+^ Tregs significantly inhibited the proliferation of CTL and IFN‐γ secretion (92). Single‐cell RNA sequencing revealed high levels of TNFR2^+^ tumor-infiltrating Tregs in gastric cancer. *In vitro* studies revealed that TNFR2 increased the FOXP3 expression in Tregs and promoted TGF‐β production, which thus enhanced the immunosuppressive phenotype of Tregs (93). It was shown that human TNFR2-specific antagonistic antibodies can inhibit the proliferation of Tregs and promote the expansion of Teffs (94).

In a preclinical study, the anti-TNFR2 antibody BI-1808 demonstrated potent antitumor efficacy in CT26, MC38, and B16 cancer models as a single agent or in combination with an anti-PD-1 antibody. BI-1808 depleted early tumor-infiltrating Tregs and induced promotion of Teffs at the tumor site, which improved the ratio of CD8^+^ Teffs to Tregs and tumor regression (95). Currently, BI-1808 is being studied in Phase I/II clinical trials. Another antibody targeting TNFR2, LBL019, has also entered clinical studies for the treatment of an undisclosed solid tumor.

### Targeting cytokine receptors

The cytokine receptors that are highly expressed on Tregs drive the migration ability of Tregs, which is a key factor to regulate tissue inflammation. One exception is CD25, which inhibits Teff activation by consumption of IL-2 in the environment. TCR-trafficking studies showed that a large proportion of tumor-infiltrating Tregs are derived from the peripheral circulation. Therefore, targeting cytokine receptors and impeding Treg migration into TME should be a primary consideration to induce a beneficial antitumor immune response in patients with solid cancer.

*CD25*

CD25, a high-affinity receptor alpha chain of IL-2Rα, was the first surface marker used to identify and isolate Tregs prior to the discovery of FOXP3 (46). It is also the most extensively studied target for depleting Tregs. Whereas CD25 is constitutively expressed at high levels on Tregs and absent on naïve Teffs, transient upregulation has been described upon activation of Teffs (96).

Using anti-CD25 antibodies with enhanced binding affinity to FcγRs led to effective depletion of tumor-infiltrating Tregs, which increased the ratio of Teffs to Tregs and enhanced the control of established tumors (97). RG6292, an anti-CD25 antibody, is optimized to deplete Tregs whilst preserving IL-2-STAT5 signaling on Teffs. In both non-human primates and humanized mouse models, efficient Treg depletion by RG6292 was observed with no overt immune-related toxicities (98). This antibody is in a Phase I clinical trial. These studies demonstrated CD25 as a therapeutic target and promising substrate in immune oncology.

As a proof of concept, camidanlumab tesirine, a DNA-damaging toxin that is based on pyrrolobenzodiazepine (PBD) dimer and targeting CD25, is the first antibody-drug conjugate (ADC) as immunotherapeutic agent to directly target immune cells rather than tumor cells. Camidanlumab tesirine is able to deplete Tregs and eradicate established tumors *via* antitumor immunity (99). In a Phase I clinical study, camidanlumab tesirine has been shown to be effective in treating Hodgkin’s lymphoma, with an overall response rate (ORR) of 86% and a complete response rate (CR) of 49% in patients with an acceptable safety (100). Another ADC, RM-1995, is composed of an antibody targeting CD25 and a photoactivating dye (IRDye^®^ 700DX [IR700]) that could be activated by illumination with 690 nm nonthermal red light (101). The RM-1995 photoimmunotherapy was designed to specifically kill CD25^-^ Tregs within solid tumors once activated with illumination.

*CCR4*

The CC chemokine receptor 4 (CCR4) is expressed on Tregs and other Th cells and binds to its two ligands CCL22 and CCL17. It is a key chemokine receptor in mediating Treg trafficking into TME (102–104) and has long been regarded as a potential therapeutic target for allergic diseases and tumor.

In addition, 80% and 73% of Tregs express cutaneous lymphocyte antigen (CLA) and CCR6, respectively, which is the phenotype of skin-homing T cells (105). Mice with CCR4^-/-^ Tregs developed lymphocyte infiltration and severe inflammatory disease in the skin and lungs (106). These findings suggest that elimination of CCR4^+^ Tregs may severely disturb skin homeostasis.

Mogamulizumab is a fully humanized, defucosylated anti-CCR4 antibody that was approved by FDA in 2018 for the treatment of relapsed/refractory cutaneous T-cell lymphoma. Mogamulizumab has a high affinity for the N-terminus of CCR4. Once bound to CCR4, mogamulizumab exerts potent antitumor effects through ADCC, inducing cell-mediated lysis and depletion of CCR4-expressing malignant T cells and Tregs (107).The therapeutic efficacy of Mogamulizumab on adult T-cell leukemia and cutaneous T-cell lymphoma is also considered to be partly due to depletion of immunosuppressive Tregs (108, 109).

*CCR8*

CCR8 is a chemokine receptor that has recently been identified as a potential specific marker for tumor-infiltrating Tregs (110) and a core member of the interferon regulatory factor 4 (IRF4)-dependent “effector” Treg gene program (111). CCR8 expression marks highly active Tregs featuring the highest levels of inhibitory receptors. Researchers compared the RNA-seq data of Tregs in tumor tissues, normal tissues, and peripheral blood of breast cancer patients and found that Tregs at the tumor site had a high CCR8 expression (112).

CCR8^-/-^ mice did not have altered numbers and proportions of Tregs in the tumor and spleen, nor did CCR8 deletion affect the growth of MC38-transplanted tumors. These results indicate that CCR8 does not influence the migration ability and function of Tregs in TME (113). Therefore, the mechanism of CCR8-targeting therapeutic antibodies for cancer may rely on Fc region-mediated ADCC and ADCP.

On another equally important note, 40% of peripheral blood CCR8^+^ Tregs are phenotypically similar to tumor-infiltrating CCR8^+^ Tregs and share many TCR clonotypes (114, 115), which suggests that tumor-specific Tregs recirculate between the tumor tissue and blood compartments, providing a rationale for targeting CCR8 on circulating Tregs.

In comparison to CCR4, T cells expressing CCR8 are much less common, being primarily restricted to skin T cells and tumor Tregs. Based on these considerations, it is possible that CCR8-directed antibodies may be better tolerated in patients and have acceptable safety profiles.

At present, there are 5 therapeutic antibodies targeting CCR8 in clinical studies. All of them eliminate CCR8^+^ Tregs through the action of ADCC or ADCP to exert antitumor effect.

### Novel approaches to targeting intratumoral Tregs by bispecific antibodies

Since Tregs lack specific targets, researchers are developing dual- or triple-targeted antibodies binding receptors that are highly expressed on tumor-infiltrating Tregs (116, 117). Compared to monoclonal antibodies, bispecific antibodies have two main advantages: Firstly, compared to monoclonal antibodies, bispecific antibodies have stronger specificity and targeting, which can reduce off-target toxicity and improve drug safety. Secondly, compared to combination therapy with monoclonal antibodies, dual- or triple-targeted antibodies can also effectively reduce the cost of drug development, clinical trials, and treatment. For example, PD-1 supports tumor immune evasion and increases FOXP3 expression *in vitro* (118, 119). However, since tumor-infiltrating CD8^+^ T and Tregs express PD-1 at equivalent high level, blocking this signaling pathway will not only activate CD8^+^ Teffs, but also enhance the immunosuppressive activity of Tregs (22). Therefore, anti-PD-1 antibody is usually used in combination with Treg-depletion antibody to increase the ratio of Teffs to Tregs and achieve better antitumor effect. Nowadays, there are several bispecific antibodies that simultaneously target PD-1 and a marker of Tregs, such as CTLA4 (Cadonilimab) (120), LAG3 (Tebotelimab and RG-6139), TIM3 (RG-7769), TIGIT (AZD2936), and CD25 (IBI-363) (**Table 2**).

**Table 2:** Next-generation bispecific antibodies targeting Tregs in tumor immunotherapy.

Drug name	Target	IgG Type	Cancer type	Company	Year of approved/clinical phase
Cadonilimab	CTLA4, PD-1	IgG1	cervical cancer,	Akeso	2022
Tebotelimab	LAG3, PD-1	IgG4	Breast, endometrial, esophageal, gastric head and neck Cancer	MacroGenics	Phase 2/3 NCT04082364
RG-6139	LAG3, PD-1	–	Esophageal, liver, non-small-cell lung cancer, melanoma	Roche	Phase 2 NCT05419388
AZD-7789	TIM3, PD-1	–	Advanced solid tumor, hodgkins disease, metastatic non-small-cell lung cancer	AstraZeneca	Phase 1/2 NCT05216835
RG-7769	TIM3, PD-1	–	Esophageal, non-small-cell lung Cancer, malignant melanoma	Roche	Phase 2 NCT04785820
AZD-2936	TIGIT, PD-1	–	Non-small-cell lung cancer	AstraZeneca	Phase 1/2 NCT04995523
IBI-363	CD25, PD-1	–	Advanced solid tumor, lymphoma	Innovent Biologics	Phase 1 NCT04995523
ATOR-1015	OX40, CTLA4	IgG1	Melanoma, metastatic colorectal, cancer, metastatic renal cell carcinoma	Alligator	Phase 1 NCT03782467
ATOR-1144	GITR, CTLA4	IgG1	Solid tumors, hematological malignancies	Alligator	Preclinical
INV-322	CD25, CTLA4	IgG1	Solid tumor	Invenra	Preclinical

"-" indicates that no relevant information was found.

In addition to PD-1, CTLA4 is also a popular target of bispecific antibodies. ATOR-1015 is a human CTLA4- and OX40-targeting IgG1 bispecific antibody generated by linking a natural CTLA4 ligand, the Ig-like V-type domain of human CD86, to an agonistic OX40 antibody. ATOR-1015 treatment reduced the frequency of Tregs and induced the activation of CD8^+^ T cells, which reduced tumor growth and improved survival in bladder, colon and pancreas cancer models (121). Furthermore, a novel GITR×CTLA4 bispecific antibody ATOR-1144 has shown to deplete Tregs *in vitro* through ADCC mechanisms (122). Moreover, the bispecific antibody INV322, engineered using Invenra’s fully human B-Body^®^ platform, co-targets CTLA4 and CD25. *In vitro* studies have characterized avidity-mediated binding of INV322 to these two tumor Treg antigens, as well as ADCC-mediated mode of action. *In vivo* studies using a murine surrogate also showed potent antitumor responses after a single dose and selective Treg depletion in syngeneic mice (123). These preliminary data demonstrate the potential of bispecific antibodies against multiple targets expressed on Tregs.

## Conclusion and prospect

Tregs correlate with poor prognosis in solid cancers, such as non-small-cell lung cancer, ovarian cancer, gastric cancer, and melanoma (110), which signifies the importance of targeting Tregs in cancer therapy. Tregs play several immunosuppressive roles in the tumor microenvironment, not only inhibiting the function and activation of T cells, APC and NK, but also secreting some immunosuppressive factors such as IL-35, IL-10 and TGF-β. Otherwise, some researchers support the longstanding hypothesis that Tregs play an important immunosuppressive role in the failure of immune checkpoint inhibitor-based immunotherapy in clinical trials, especially for many patients who have no response after anti-PD-1 treatment. Part of the reason is that anti-PD-1 can not only activate T cells, but also activate Tregs, making Tregs have stronger immunosuppressive activity (26).Therefore, regulating the proportion of Tregs or their signaling pathways in TME to reverse the immunosuppressive microenvironment may have a positive effect on the treatment by immune checkpoint inhibitors. Treg is targeted first and then combined with T cell engagers, better therapeutic effects may be achieved.

Nearly a century has passed since Tregs were discovered to play a role in self-tolerance and homeostasis distinct from conventional T cells. So far, the immunosuppressive effects of Tregs in cancer have been well investigated, with several studies affirming the difference between peripheral blood and tumor-infiltrating Tregs. But there are still many issues in targeting Tregs to treat cancer and autoimmunity. One main open question is how to control Tregs to augment tumor immunity without influencing autoimmunity, or to suppress the latter without inhibiting the former. A better understanding of the heterogeneity and function of Tregs in autoimmunity and cancer may help to find solutions. Many studies have concluded that there are barely specific targets on Tregs, and most of the targets of tumor-infiltrating Tregs are expressed on Teffs or NK cells in TME, especially for immune checkpoint molecules and co-stimulatory molecules. Targeting these molecules has the potential to equally damage effector cells. For example, mogamulizumab administered to patients in clinical studies may not differentiate between targeting effector Tregs and central memory CD8^+^ T cells, especially at high doses (124) However, currently, most antibodies used to eliminate Tregs increase rather than decrease the proportion of CD8^+^ T cells while eliminating Tregs, such as RG6292 (98). The reason may because that the expression level of these targets on effector T cells is much lower than tumor infiltrating Tregs. However, this cannot rule out that some Teffs are cleared by antibodies (98). Many researches have been evaluated to address this issue. Some novel immunotherapies target two or more markers on tumor-infiltrating Tregs, such as CTLA4/OX40 or CTLA4/PD-1, which improves specificity and reduces off-target effects, or adjusting the affinity of the antibodies so that they bind weakly to low-density targets and strongly to high-density targets, another method is to adjust the dosage to avoid excessive killing of Teffs. In addition, some ADC-based therapies directly kill Tregs through toxin to fix the insufficient ADCC effect caused by the exhaustion of Teffs in TME.

Another important question in tumor immunity is how to eliminate tumor-infiltrating Tregs without affecting Tregs in the peripheral blood. Compared to Tregs in the peripheral blood, intratumoral Tregs exhibit a more proliferative and immunosuppressive phenotype and are characterized by elevated expression of CTLA4, CD25, GITR, OX40, LAG3, TIM3, TIGIT, CCR4, CCR8, and PD-1. The affinity of antibodies to these targets should be carefully considered when screening antibodies. Antibodies with high affinity may cause disorder of peripheral Tregs, while antibodies with low affinity may not achieve the desired immunotherapy outcome and cause off-target effects. For instance, RG-6139 is a bispecific antibody that targets both LAG3 and PD-1. It has a higher affinity for PD-1 than for LAG3, which allows the bispecific antibody to target activated tumor-infiltrating T cells while effectively targeting Tregs.

The biological function of Tregs in TME is complex, and there are still many issues to be solved in the future to develop new treatment and immune-precision drugs for each cancer patient. Whether the next generation of drugs targeting Tregs can further improve patient outcomes while maintaining tolerance and particularly low risk of autoimmunity will be of paramount importance. It is hoped to make cancer immunotherapy more effective with fewer side effects in the future.

## Author contributions

QL, YW and TY conceived and wrote the paper. JLu, J.Li and BZ revised and edited the final version of the manuscript. All authors contributed to the article and approved the submitted version.
